# Securing peripheral intravenous catheters in babies without applying adhesive dressings to the skin: a proof-of-concept study

**DOI:** 10.1186/s12887-022-03345-8

**Published:** 2022-05-18

**Authors:** Deborah L. Harris, Melissa Schlegel, Anna Markovitz, Lisa Woods, Tamara Miles

**Affiliations:** 1 School of Nursing, Midwifery and Health Practice, Faculty of Health, Victoria University of Wellington, PO Box 7625, Newtown, Wellington 6242 New Zealand; 2 Newborn Intensive Care Unit, Capital Coast District Health Board, Wellington, New Zealand; 3 Waikato District Health Board, Hamilton, New Zealand; 4 School of Mathematics and Statistics, Victoria University of Wellington, Wellington, New Zealand

**Keywords:** Infant, Newborn, Neonatal Intensive Care Unit, Iatrogenic Skin Injury, Splints

## Abstract

**Background:**

Most babies admitted to a Neonatal Intensive Care Unit (NICU) require a peripheral intravenous catheter (PIVC). PIVCs are secured using splints and adhesive dressings applied to the skin. Removing the dressings causes skin injury, pain, and risks infection. We designed the Pēpi Splint, which supports PIVCs without the application of adhesive dressings to the skin. We sought to determine the effectiveness and acceptability of the Pēpi Splint using a proof-of-concept design.

**Methods:**

Eligible babies were > 1000 g and > 30 weeks’ corrected gestation admitted to Wellington Regional NICU and who required a PIVC. All babies received the same care as those not in the study, with the addition of the Pēpi Splint. Primary outcomes were the proportion of babies in which the Pēpi Splint secured the PIVC for the required time and proportion of babies who experience an adverse event. Secondary outcomes were the acceptability of the Pēpi Splint as reported by the parents.

**Results:**

Thirty-eight babies, median (range) birth weight 2625 g (396—4970) and gestation 37wk (22—41). When the Pēpi was applied the postnatal weight was 2969 g (1145 – 4970) and gestation 37wk (29 – 41). The Pēpi Splint held the PIVC secure for 34/38 babies (89%), for a duration of 37 h (6 to 97). There were no adverse events. Of the four babies reported to have unsecure PIVCs, two were due to the securement two were displaced during feeding. Fifty-eight parents responded to a questionnaire (32 mothers, 26 fathers). Of these parents 52 (90%) would participate again and 52 (90%) would recommend participating to others. Overall, clinicians reported the Pēpi Splint was easy to use 33/38 (87%).

**Conclusion:**

The Pēpi Splint safely secures PIVCs without adhesive dressings being applied to the skin and is acceptable to both parents and clinicians. Our findings provide support for a larger multicentred randomised controlled trial.

**Trial registration:**

Registered with the Australian and New Zealand Clinical Trials Registry Reference ACTRN12620001335987.

## Background

Admission to a Neonatal Intensive Care Unit (NICU) is not uncommon. Since the establishment of NICUs admission rates have increased and neonatal mortality rates for preterm and unwell babies have fallen, which is attributed mainly to the specialist care provided by the clinical teams and improvement in the technology and equipment [1]. Babies who are admitted to NICU require multiple invasive procedures, interventions which are fundamental to providing treatment and improving clinical outcomes. The most common procedure is the placement of a peripheral intravenous catheter (PIVC). These catheters provide necessary vascular access for fluids, nutrition, medications, and blood products. However, PIVCs are associated with a high risk of iatrogenic injury, including extravasation, infiltration, catheter related blood infections and scarring [2, 3]. PIVCs can be difficult to insert and once inserted the duration of the PIVC is reasonably short, and commonly two to three days [4] Therefore, many babies require repeated catheters for the duration of hospitalization [3].

PIVCs must be well secured to reduce the risk of catheter failure, extravasation and injury. It is recommended that the PIVC is secured with a splint or board on the limb to adequately immobilize the joint and reduce the risk of venous damage resulting from flexion of the joint [5]. However, extravasation injuries are common as babies have poor venous and epithelial integrity [4, 6]. Extravasation injuries include pain, infiltration, phlebitis, infection, and in extreme cases, skin sloughing and scarring [2, 4]. Visibility to the PIVC insertion site is essential to allow clinical staff regular observation and prompt treatment if redness or swelling is noted to reduce the incidence of these injuries [7].

Splints are secured to the baby’s skin using a variety of methods, but most commonly adhesive dressings. The removal of the adhesive dressings is a common cause of epidermal stripping. A single adhesive removal has been shown to strip 70–90% of a baby’s epidermis (though multiple adhesive replacements can cause deeper injury), as the adherence of tape-to-skin is often stronger than the adherence of skin layers to each other [8]. Skin injuries are common in the NICU and the majority are unreported, of those reported most are related to the devices used by clinicians to provide patient care [9].

Following a traumatic skin injury which occurred during the removal of adhesive dressings. We sought alternative methods to secure intravenous catheters without the need for adhesive dressings to be applied to the babies skin. We collaborated with a design engineer to create a new splint: The Pēpi Splint is a non-invasive, non-sterile device, it is likely to fall into the lowest risk category for most jurisdictions [10] and was registered with the Medical Devices Safety Authority (Medsafe) and the WAND database on 19 July 2018. The patent registrations are as follows European Patent Application No. 19799163.1, Canadian Patent Application No. 3099939; United States of American Patent Application No. 17/054,649; Australian Patent Application No. 2019267139; PCT Application No. PCT/NZ2019/50052.

The Pēpi Splint is made from PlatSil® Silicone gel, a product commonly used in prosthetics, and used to treat cleft lip scarring in babies [11]. Within the silicone is a non-magnetic aluminium, which allows the Pēpi Splint to be moulded to the baby’s limb and provides the opportunity for the splint to remain in place during magnetic resonance scanning (MRI). We cannot find any evidence of harm caused to babies due to the use of silicone gel. The Pēpi Splint seeks to secure the PIVC and eliminate the need for adhesive dressings to secure the splint on to the baby’s skin entirely, as the adhesive tapes are applied only to the Pēpi Splint itself. Further, the insertion site of the PIVC can remain visible, allowing clinical staff to review the PIVC insertion site. It weighs approximately 45 g and can be washed and sterilized for reuse.

We sought to determine the effectiveness and acceptability of the Pēpi Splint to both clinicians and parents using a proof-of-concept pilot study. We were particularly interested to understand if the newly designed splint would secure the PIVC for the required duration, the incidence of any skin injury, and acceptability of the Pēpi Splint to the clinical staff and parents.

## Methods

Our study was a proof of concept, prospective intervention study. Eligible babies were > 1000 g and > 30 weeks’ gestation admitted to Wellington Regional NICU and who required a PIVC. Written informed consent was collected by parents prior to participation.

**Fig. 1:**
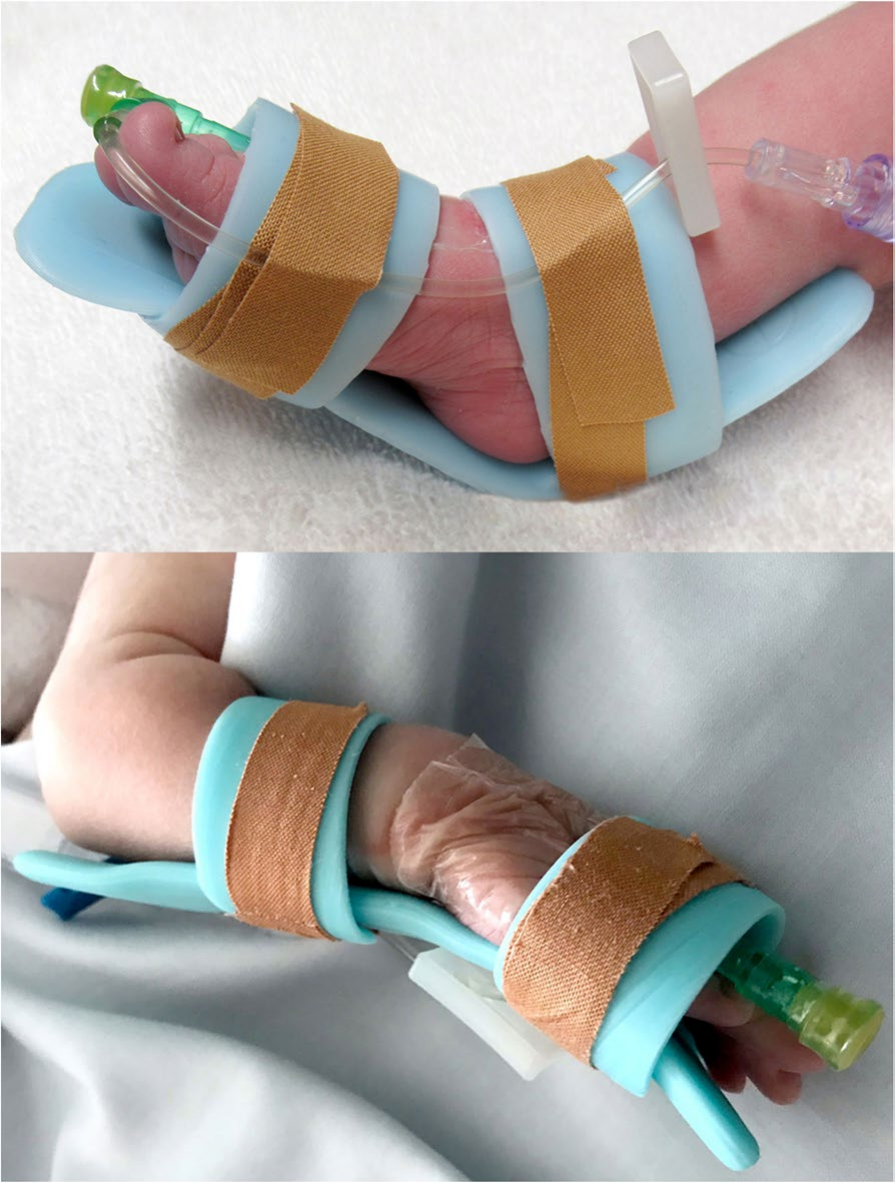
The Pēpi Splint securing a peripheral intravenous catheter on a hand and foot

Clinical staff inserted a PIVC into the baby’s hand, arm, foot or leg and secured it using a 3 M Tegaderm Transparent dressing [5]. A Pēpi Splint was then selected from one of three available sizes (small, medium, large > 3000 g; single use only) and applied to the baby. Elastoplast adhesive dressings were then applied directly to the Pēpi Splint (as opposed to the skin of the baby) to fully secure the PIVC (Fig. 1). Routine clinical care for babies with PIVCs includes hourly observation and documentation by the bedside nurse. All babies received the same care as those not in the study but with the addition of the Pēpi Splint. After the removal of the Pēpi Splint the baby returned to routine clinical care.

The clinical team (a clinical nurse specialist, nurse practitioners, and a neonatologist) collected data on whether the Pēpi Splint was easy or difficult to apply and remove. The time that the Pēpi Splint was applied and removed. Whether the Pēpi Splint secured the PIVC. The reason for PIVC and Pēpi Splint removal, along with concerns (e.g., skin injury). Standardized photos were taken of the Pēpi Splint following application. These images included a white tape measure to allow for colour correction and measurement of any injury [12]. The primary outcomes were the proportion of babies in which the Pēpi Splint was judged by the clinical team to have supported the PIVC and the proportion of babies who experienced an adverse event related to the Pēpi Splint. Secondary outcomes were the acceptability of the Pēpi Splint, as determined by the bedside clinician and parents. An adverse event was any skin damage attributed to the Pēpi Splint by a senior clinician within the NICU.

Data collected from parents included a short questionnaire administered by the research nurse. The questionnaire was designed from a previously published questionnaire [13]. Parents were asked what they liked and disliked about the Pēpi Splint, such as whether they thought it adequately secured their baby’s PIVC, as well as their general experiences participating in the study. Secondary outcomes were the acceptability of the Pēpi Splint, as determined by the bedside clinician and parents.

### Modifications to the Pēpi Splint

During the initial phase of the study, some clinicians reported the Pepi Splint to be challenging to use and parents reported that the PIVCs were not secure. Clinicians were frequently required to adjust the adhesive taping (n = 7). In response, the principal investigator and the clinical team stopped recruitment while all reports and photos from each participant were reviewed. The Pēpi Splint was reported to slip during use therefore the PIVC was less secure, causing a lack of confidence about the Pēpi Splint itself. After discussion with the design team, the PlatSil® Silicone gel mixture was altered to include PlatSil® Prosthetic Deadener, making products feel more skin-like. Plus, small ridges were included in the internal aspect of the Pēpi Splint. In addition, further education was provided to the clinical team about how to secure the Pēpi Splint.

### Statistical analysis

This study was a proof-of-concept design to determine if the Pēpi splint was effective in supporting the PIVC and did not cause harm. If the splint was found to be effective and safe, the findings from this study would allow for progression to a multisite randomised control trial seeking to determine superiority of the Pēpi splint compared to standard care [14]. Within the proof-of-concept study we sought to determine no more than 10% of babies would experience an adverse event (skin injury attributed to the Pēpi Splint). We estimated with a sample size of 29 babies there was 97% power to determine the rate of adverse events to be < 10% in the population (assuming that an adverse event occurs one in 1000 babies).

Two exact 95% confidence intervals were calculated: one for the proportion of babies in which the Pēpi Splint was judged by the clinical team to have secured the PIVC and one for the proportion of babies who experienced an adverse event. Descriptive statistics are presented for categorical data where appropriate. All data were independently entered by two investigators and later compared for agreement by the research nurse. Variations between data entries were compared and discussed. Statistical analyses were performed with JMP v14 and R v 3.6.1. The protocol was designed using the SPIRT guidelines [15, 16] and is available online https://openaccess.wgtn.ac.nz/articles/report/The_P_pi_Splint_Project_Protocol/16767193

Ethical approval was granted from the New Zealand Health and Disability Ethics Committee.

Central Ethics Committee 20/CEN/47. The Trial is registered with the Australian and New Zealand Clinical Trials Registry Reference ACTRN1262000133598, 11/12/2020. An external safety monitoring committee (harms) was established and defined an adverse event as skin damage (i.e., skin irritation or injury or pressure areas) attributed to the use of the Pēpi Splint.

## Results

Thirty-eight babies were enrolled in the Pēpi Splint Study (Table 1). Clinicians reported the Pēpi Splint secured the PIVC for 34/38 (89%) babies, for a median duration of 37 h (range 6 to 97).

**Table 1:** Characteristics of babies

**Baby (*n* = 38)**	
At birth	
Gestation (weeks)	37 (22—41)
Birthweight (g)	2625 (396—4970)
Male	24 (63)
*At the time the Splint was applied*	
Gestation (weeks)	37 (29—41)
Weight (g)	3011 (1145—4970)
Reason for admission to Newborn Intensive Care Unit	
Respiratory Distress	14 (37)
Prematurity	12 (32)
Surgery or Investigations	7 (18)
Hypoglycaemia	5 (13)
Ethnicity	
New Zealand European	18 (48)
Māori	7 (18)
Indian	4 (11)
Pacific	2 (5)
Other^a^	7 (18)

Data are mean (SD), median (range), number (%)

^a^Other means European (4) and Filipino (2) Sri Lankan (1)

No adverse events were reported. Initially the clinicians reported the Pēpi Splint to be challenging to use. However, following modification of the Pēpi Splint, clinicians reported that the product was easy to use (Table 2). There were four individual reports from the bedside nurse that the Pēpi Splint did not secure the PIVC: two reported the taping around the Pēpi Splint caused the PIVC to become unsecure, which was corrected with adjustment of the adhesive tape; and two PIVCs were dislodged during movement of the baby from the cot for breastfeeding.

**Table 2:** Reported clinical outcomes from the Pēpi Splint study

	Total Babies (*n* = 38)	Babies with original Splint (*n* = 7)	Babies with modified Splint (*n* = 31)
**Primary outcomes**			
Secured the PIVC for the required time	34 (89)	5 (71)	29 (93)
Skin injury related to splint	nil	nil	nil
Application of the Pēpi Splint			
Reported to be easy to apply	33 (86)	2 (29)	31 (100)
Duration of use (h)	6.5 – 97.2 (36.1)	13.3 – 45.0 (23.0)	6.5 – 97.2 (41.0)
Removed due to			
No longer needed	22 (58)	4 (57)	18 (59)
Extravasation	11(29)	2 (29)	9 (29)
PIVC dislodged during feeding	2 (5)	nil	2 (6)
Leaky PIVC	2 (5)	nil	2 (6)
Concern about the splint	1 (3)	1 (14)	0
Parents withdrew from the study	nil	nil	nil

Data are presented as number (%), median (range)

PIVC means peripheral intravenous catheter

Parents largely liked participating in the study, as nearly 80% reported enjoying contributing to the improvement of health care. Parents also liked the Pēpi Splint itself, as most of them reported they liked how soft the Pēpi Splint was against their baby’s skin and that the Pēpi Splint reduced the need for adhesive dressings on the baby’s skin (Table 3). The majority of parents (52/58, 90%) reported they would participate in the study again if they had another eligible baby. Most parents (52/58, 90%) reported they would recommend participating in the study to family and friends. Dislikes about the Pēpi Splint were uncommon with10 (17%) parents reporting the Pēpi Splint did not secure the PIVC. Largely, these reports were early in the study. Three (5%) of the parents reported no reduction in the number of adhesive dressings on the baby’s skin. Further, some parents found that the Pēpi Splint made it difficult to put the baby’s clothes on. However, this is an ongoing concern with all devices used in the NICU environment and not isolated to the Pēpi Splint itself. Comments from the parents included:

*“It was good for my baby skin and I like that, but it needed to be retaped’*

*‘Really liked no tapes.”*

*“It was great to participate in the study. I wished that we could have continued to use the study splint, as one of our girls had real trouble with her skin.”*

**Table 3:** Parents reported experience of the Pēpi Splint

Completed questionnaires	Mother *n* = 32	Father *n* = 26	Total *n* = 58
**What I liked about participating in the Pēpi Splint Study**			
The Pēpi Splint itself	22 (69)	16 (62)	38 (66)
Contributing to improving health care for babies	26 (81)	20 (77)	46 (79)
Other	3 (9)	3 (12)	6 (10)
**What I did not like about participating in the Pēpi Splint Project**			
The Pēpi Splint itself	2 (6)	0	2(3)
The experience of participating	0	0	0
Other	2 (6)	3 (12)	5 (9)
**What I liked about the Pēpi Splint**			
The PIVC was secure	12 (38)	12 (46)	24 (41)
The Pēpi Splint was soft on my baby’s skin	24 (75)	20 (77)	44(76)
Reduced adhesive dressings on my baby’s skin	25 (78)	18 (69)	43 (74)
**What I did not like about participating in the Pēpi Splint Project**			
The PIVC was not secure	5 (16)	5 (19)	10 (17)
The Pēpi Splint harmed my baby’s skin	0	0	0
No reduction in adhesive dressings	1 (3)	2 (8)	3 (5)
**If I had another eligible baby, I would participate again**			
Yes	28 (88)	24(92)	52 (90)
No	4 (12)	1 (4)	5 (9)
Unsure	0	1 (4)	1 (2)
**I would recommend the Pēpi Splint to family/whānau and friends**			
Yes	29 (91)	23 (88)	52 (90)
No	2 (6)	2 (8)	4 (7)
Unsure	1 (3)	1 (4)	2 (3)
**My experience in the Pēpi Splint Study has made me more or less likely to participate in future research**			
No change	17 (53)	18 (69)	35 (60)
More likely	14 (44)	8 (31)	22(38)
Less likely	1 (3)	0	1 (2)

Data are number (%)

18 babies had one parent respond

20 babies had both parents respond

## Discussion

We have shown the Pēpi Splint to be effective in securing PIVCs for babies who need treatment and are admitted to a NICU. There were no reported skin injuries, which may be due to the absence of adhesive dressings applied directly to the skin. Both clinicians and parents found the Pēpi Splint acceptable. Clinicians reported that the Pēpi Splint secured the PIVC for the required duration. Most parents reported that they liked the Pēpi Splint and would participate again if they had another eligible baby and would also recommend participating in the study to others.

Skin injuries are common in hospitalized babies and most are underreported [12] However, evidence of reported injuries shows between 68 to 90% of all skin injuries in hospitalized newborns can be linked with the fragile skin physiology and the combination of necessary mechanical devices for treatment [17]. Despite PIVCs being the most common medical device in the NICU. There is limited evidence about the routine use PIVCs in the neonatal population. Our initial findings, show that the Pēpi Splint provides the opportunity to safely secure a required PIVC without adhesive tapes being applied to the skin. Therefore, using of the Pēpi Splint may reduce in the incidence of skin injuries for hospitalized babies.

Our findings show that the Pēpi Splint held the PIVC in place for a mean duration of 41 h, with over half of the PIVCs removed due to no longer being required and one-third of the PIVCs removed due to extravasation. Comparison with other studies are difficult due to differing methodologies. However, authors from larger studies have reported similar durations of 1- 2 days for PIVC use, and a similar frequency of complications including extravasation injury in neonatal populations [4, 18]. Of the four reports from bedside nurses which reported the PIVC to be unsecure due to the Pēpi Splint two were related to the new method of taping the adhesive dressings to the Pēpi Splint itself. Following retaping the PIVCs were reported to be secure. Clinical staff were provided with education about the best way to secure the Pēpi Splint and they were learning how to use it. It is likely that with more experience with the Pēpi Splint concerns related to securing with taping would discontinue.

The importance of the parent’s voice in the development and leadership of clinical research cannot be underestimated [19]. A member of the Pēpi Splint Steering Committee is a mother who experienced having a late preterm baby in the NICU. We asked parents what they liked and disliked about the Pēpi Splint itself along with what they liked and disliked about participating in the study. We were also interested to understand more about if participating in this study influenced likely involvement with clinical research in the future. Most parents found the Pēpi Splint to be acceptable, with nearly 80% of the parents reporting liking the softness of the Pēpi Splint and the lack of adhesive tapes on the skin of their baby. Nearly 20% of the parents were concerned that the PIVC was not secure. However, this could be mitigated with improved taping. Parents were aware that the purpose of the study and one father wrote in the comments that future research into the Pēpi Splint was warranted. Therefore, our findings signal that most parents who participated found the Pēpi Splint acceptable for use.

The initial investigation for the development of the Pēpi Splint arose following an injury. Following a serious incident, the investigation determined that the currently available products for securing PIVCs were not meeting the clinical needs of the babies within our NICU. We sought the collaboration of a design engineer which resulted in the development of the Pēpi Splint. While collaboration between nurses and design engineers are few, we provide evidence that collaboration between neonatal clinicians and medical device designers is an essential pathway to reducing skin injury. Therefore, collaboration between the health care professionals and engineering should be encouraged within clinical and university settings.

As our study was a proof-of-concept design in a single NICU. These findings require replication to provide generalizability across Newborn Intensive Care settings. However, we have provided the evidence needed to inform the design of future studies to further determine the effectiveness and potential superiority of the Pēpi Splint. Suggestions to be included for future investigations include determining: (1) the safety and effectiveness of the Pēpi Splint in different NICU settings, and with differing gestational ages, including extremely preterm babies (< 30 weeks’ gestation and < 1000 g), as these babies remain in the NICU for long periods and require numerous PIVCs, (2) the ability of the Pēpi Splint to remain in place during magnetic resonance imaging (of note, three [8%] of the babies who participated in the study required MRI scanning), (3) the opportunity for reusable Pēpi Splints.

Our findings provide the first step in changing PIVC protocols. A possible randomized control trial could compare the Pēpi Splint with the routine clinical practice, thereby determining the superiority of the Pēpi Splint more over existing medical device-securing tapes and splints. Plus, identify any significant barriers in changing practice. Our proof-of-concept study has shown that it is possible to secure PIVCs without the need for any adhesive dressings to be applied to the skin and, therefore, substantially reducing the risk of iatrogenic skin injury in newborn babies. Furthermore, an adaptation of the Pēpi Splint could be investigated for use in other at-risk populations, including young children and the elderly.

## Data Availability

The datasets used and/or analysed during the current study are available from the corresponding author on reasonable request.
